# Multifocal multi-organ ischaemia and infarction in a preterm baby due to maternal intravenous cocaine use: a case report

**DOI:** 10.4076/1752-1947-3-9259

**Published:** 2009-09-14

**Authors:** Ben C Reynolds, Dawn KM Penman, Allan G Howatson, Lesley A Jackson, Charles H Skeoch

**Affiliations:** 1 Neonatal Unit, Princess Royal Maternity Hospital, Alexandra Parade, Glasgow, UK; 2 Department of Paediatric Pathology, Royal Hospital for Sick Children, Dalnair Street, Glasgow, UK

## Abstract

**Introduction:**

Although the adverse effects of cocaine use in pregnancy are well recognised, we believe this case highlights the importance of considering the route of administration, and suggests the possibility of multifocal damage relating to intravenous use.

**Case presentation:**

A Caucasian female baby of 29-weeks' gestation was spontaneously delivered and subsequently developed multi-organ failure considered unrelated to simple prematurity. Intensive care was re-orientated following the development of massive intraventricular haemorrhage.

**Conclusion:**

This case illustrates the need for regular cranial ultrasound in babies of pregnancies at risk due to intravenous cocaine use and also the necessity of counselling women who misuse cocaine in the antenatal period. As such, this article will be of most interest to paediatric and obstetric staff.

## Introduction

Cocaine use in pregnancy has been associated with adverse fetal outcomes including congenital malformations. We report a female baby of 29 weeks' gestation whose mother had extensive polydrug misuse throughout her pregnancy, including the use of intravenous cocaine. Following spontaneous delivery, the baby died after three days of intensive support. A post-mortem examination revealed widespread ischaemic change throughout multiple organs. We hypothesise that the unusual extent of this damage is related to the route of administration and dosage of cocaine during the pregnancy.

## Case presentation

A 29-year-old Caucasian primigravida presented at 29^+0^ weeks' gestation with abdominal pain and fever. A presumptive diagnosis of urinary tract infection was made with laboratory investigations demonstrating a raised C-reactive protein and peripheral leukocytosis, and treatment with intravenous cefuroxime was commenced. The expectant mother reported regular use of heroin, diazepam, 'street' methadone and cocaine. Heroin and cocaine were both smoked and injected intravenously. Frequency of use was difficult to clarify.

Abdominal pain continued intermittently and antenatal betamethasone was administered. A cardiotocograph (CTG) trace was non-reassuring and necessitated an emergency Caesarean section approximately five hours after the initial dose of betamethasone. A female was delivered alive and in good condition, weighing 1530 g (75^th^ centile). Apgar scores were 7^1^ and 8^5^. There were no external dysmorphic features, organomegaly, rash or bleeding. An initial cranial ultrasound scan was normal with no evidence of haemorrhage. Mean blood pressure (BP) was normal. Laboratory investigations demonstrated marked coagulopathy and abnormal liver function tests (Table 1). Aspartate transaminase (AST) was disproportionately elevated in comparison with other liver enzymes, a pattern suggesting extensive tissue injury due to the non-specificity of AST.

**Table 1:** Temporal evolution of laboratory parameters

Laboratory parameter		Age		
Parameter	Reference Range	1 hour	12 hours	36 hours
PT	10.6-16.2 secs	54	29	
APTT	27.5-79.4 secs	60	41	
TCT	19.2-30.4 secs	23	19	
Fibrinogen	1.5-3.73 g	0.5	1.2	
Urea	2.5-7.5 mmol/l		6.4	9.5
Creatinine	35-100 μmol/l		78	136
Bicarbonate	21-28 mmol/l	22.0	15.6	13.1
AST	<40 U/l		1891	2168
ALT	<50 U/l		200	203
Gamma-GT	<55 U/l		259	227
Albumin	g/l		21	17

Stated coagulation reference ranges are applicable to 30-week gestation healthy controls on the first day of life. ALT, alanine transaminase; APTT, activated partial thromboplastin time; AST, aspartate transaminase; Gamma GT, gamma glutamyl transferase; PT, prothrombin time; TCT, thrombin clotting time.

Fresh frozen plasma (FFP) and cryoprecipitate were administered without improvement in the coagulopathy. Urine was noted to be pink in colour, but microscopy did not demonstrate red cells. At 16 hours of age, there was generalised seizure activity confirmed on amplitude-integrated EEG (Cerebral Function Monitoring - 'CFM'). The infant was loaded with phenobarbitone and received a half correction of sodium bicarbonate for a progressive metabolic acidosis. Morphine was infused at 10 micrograms/kg/hour.

Urine output was <0.5ml/kg/day by 24 hours of age and she was passing extremely liquid stools. Coagulopathy persisted and liver function deteriorated further on sequential monitoring (Table 1). Repeat ultrasound at 36 hours of age showed bilateral intraventricular blood with evidence of marked midline shift. It was decided that continuing care aimed at the baby's survival was inappropriate and care was re-orientated following discussion with the baby's mother. The infant was extubated one hour following baptism, and died shortly afterwards.

**Figure 1:**
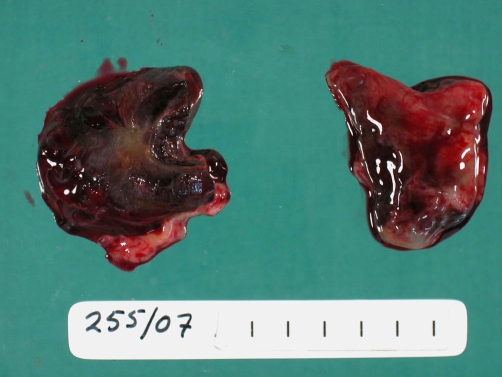
**Bilateral blood casts of cerebral ventricles**. Post-mortem pathological specimen demonstrating 'cast' formed by cerebral ventricles entirely filled with blood following massive intraventricular haemorrhage.

**Figure 2:**
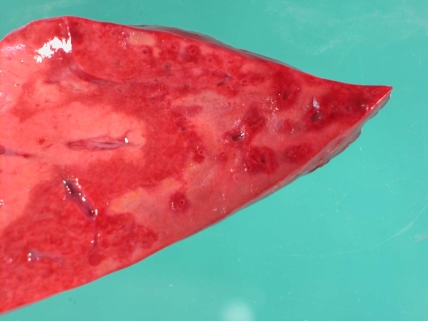
**A clear demarcation of healthy liver on the left, ischaemic liver centrally, and necrotic areas to the right**. Postmortem pathological specimen of liver demonstrating zonal multifocal necrosis, with marked macroscopic necrosis visible on right side of specimen, healthy liver on left and a 'border' of ischaemic tissue between.

A postmortem examination was performed and it demonstrated intraventricular haemorrhage (IVH) (Figure 1) expanding all four ventricles and extending around the brain stem and cerebellum (grade 3). Histology showed recent subarachnoid haemorrhage and cortical vascular congestion consistent with multiple small focal interstitial haemorrhages distinct from the IVH. There was hepatic necrosis (Figure 2) and evidence of colonic mucosal ischaemic injury with multiple punctate erythematous areas. The kidneys showed zonal interstitial haemorrhage involving the medullary pyramids. The bladder also contained an area of large submucosal haemorrhage. These urogenital changes probably explain the pink-coloured urine. The absence of red cells was possibly attributable to haemolysis within the urinary tract. In addition, there was ischaemia and necrosis of the islets of Langerhans with sparing of the exocrine pancreas. Thymus, heart and adrenals appeared normal. Examination of the placenta showed acute decidual haemorrhage and chronic intervillitis. Microbiological and metabolic investigations did not demonstrate any further cause for deterioration or death.

## Discussion

Cocaine has been used for recreational purposes for over 5000 years [1]. The drug can be ingested, smoked, injected or inhaled intranasally. Smoking and snorting cocaine are the most common methods of cocaine use. Intravenous use is infrequent and account for less than 10 percent of cocaine use in the USA [2]. Comparative figures for the UK are unavailable and comprehensive Department of Health public information only briefly mentions injection as a route of administration [3].

Adverse effects of cocaine on the adult user are well recognised [1]. Vasoconstrictive effects are mediated via blockage of catecholamine uptake and beta-adrenergic stimulation. Cocaine use during pregnancy and its teratogenic effects on the fetus are less well defined. Early observational reports suggested 'crack babies' could have a variety of congenital abnormalities, including gastroschisis, intraventricular haemorrhage, growth restriction, and genitourinary and renal anomalies [4]. While evidence has increased, meta-analyses [4] and larger scale studies [4] have not confirmed any of the anatomical sequelae, although behavioural effects appear true. The mode of cocaine use is rarely considered or controlled for, nor is the cumulative dose of cocaine. Polydrug use and the chaotic lifestyle associated with substance misuse are variably considered as confounding within studies. Maturity at birth is also often omitted though it is suggested that preterm babies are affected differently [6].

The role of cocaine in intraventricular haemorrhage is still unclear. A prospective study [7] comparing light and heavy cocaine users with controls demonstrated an increased incidence of subependymal haemorrhage within term babies in the heavy cocaine user group only. A subsequent retrospective review [8] found a similar finding in preterm babies. Although, the review did not stratify according to cocaine usage, it suggested that this effect may have been even more pronounced in mothers who used large quantities. A small prospective study [9] of very low birth weight (VLBW) babies showed a higher incidence of grade I to II haemorrhage, but not more severe bleeds. A further larger prospective study of VLBW babies [10] did not find any increased risk of grade III or IV intraventricular haemorrhage though it did not consider dosage for confounding or consider smaller bleeds.

Widespread focal ischaemia and infarction affecting multiple organs have not previously been reported in an infant as a result of maternal cocaine use. We hypothesise that the postmortem findings are related to the vasoconstrictive effects of cocaine use. The occurrence or extent of intraventricular haemorrhage within cocaine-exposed babies may be related to dosage. Intravenous usage may aggravate this effect. This case is of particular interest due to the widespread nature of the ischaemic infarcts affecting multiple organ systems. The focal nature of the infarcts affecting multiple organs makes them highly unlikely to be attributable to either complications of prematurity or the other illicit substances taken during this pregnancy. Due to the mixed nature, another substance or a cumulative effect cannot be excluded. However, similar infarcts have not, to our knowledge, been reported with heroin, methadone, or benzodiazepine use.

## Conclusions

We advocate early and regular coagulation screening and cranial ultrasound scans for pregnant women with significant cocaine use, particularly if taken intravenously. The risk of significant morbidity and mortality should be considered during antenatal counselling of women who use cocaine. We also suggest that there is a need for further prospective research in this area with dosage and mode of administration being considered as confounding factors.

## Abbreviations

ALT: alanine transaminase; APTT: activated partial thromboplastin time; AST: aspartate transaminase; BP: blood pressure; CFM: cerebral function monitoring; CTG: cardiotocograph; EEG: electroencephalogram; FFP: fresh frozen plasma; GGT: gamma glutamyl transferase; IVH: intraventricular haemorrhage; PT: prothrombin time; TCT: thrombin clotting time; VLBW: very low birth weight.

## Consent

Written informed consent was obtained from the parent of the patient for publication of this case report and accompanying images. A copy of the written consent is available for review by the Editor-in-Chief of this journal.

## Competing interests

The authors declare that they have no competing interests.

## Authors' contributions

BCR and LAJ were the principal contributors to the manuscript, and primarily involved in the care of the baby. DKMP and AGH performed the postmortem and kindly provided the figures. CHS revised the manuscript. All authors read and approved the final manuscript.
